# Supplementary material for: Hydrocarbon Resin-Based Composites with Low Thermal Expansion Coefficient and Dielectric Loss for High-Frequency Copper Clad Laminates
Source: Polymers (Basel). 2022 May 28;14(11):2200. doi: 10.3390/polym14112200 (PMC9182675; doi:10.3390/polym14112200)
Supplement: Supplementary file 1 [file polymers-14-02200-s001.zip › polymers-1727002-supplementary.pdf]

polymers

MDPI

Supplementary Materials: Hydrocarbon resin-based composites with low thermal expansion coefficient and dielectric loss for high-frequency copper clad laminates

Jiaojiao Dong, Hao Wang, Qilong Zhang, Hui Yang, Jianlin Cheng and Zhaoyue Xia

Dielectric property

At high frequency, the relative permittivity of the material is the complex number ε*:

$$\varepsilon^* = \varepsilon' - j\varepsilon'' \quad (1)$$

The imaginary part ε'' represents the loss of the material at high frequency, and δ is defined as the residual angle δ tangent of the phase angle between the applied sine wave voltage and the current passing through the substrate.

$$\tan\delta = \varepsilon''/\varepsilon' \quad (2)$$

In the experimental part of the paper, the complex number ε* was tested as Dk (dielectric constant) by an SPDR (separated dielectric resonator) and as Df (dissipation factor, also called dielectric loss) for the dissipation angle tangent.

The optimal content of crosslinker was explored, substrates with different content of DBPH were designed, and their dielectric properties were tested at high frequency. Fig. 1(a) shows that the higher the content of crosslinker, the higher the D_f. There many C=C in uncross-linked polyolefin resin. In the process of crosslinking, C=C transforms into C-C, then C-C dimers connects with each other to form a network structure, which is conducive to reducing polarization and D_f. The crosslinking efficiency can be characterized by the content of C=C in a sample. The higher the amount of C=C, the lower the crosslinking efficiency. The absorption peak of C=C is at about 1600 cm^{-1}. As can be seen in Fig. 1(c), the sample with 0 phr DBPH showed the strongest infrared absorption peak of C=C, proving that it contained the higher amount of C=C. Similarly, it was possible to infer the C=C content in other samples. It was proved that the crosslinking efficiency reached the maximum value when DBPH was added at 1.07 phr. An excessive amount of cross-linking agent causes impurities, resulting in the increase of the polarization groups and the D_f of the composites.

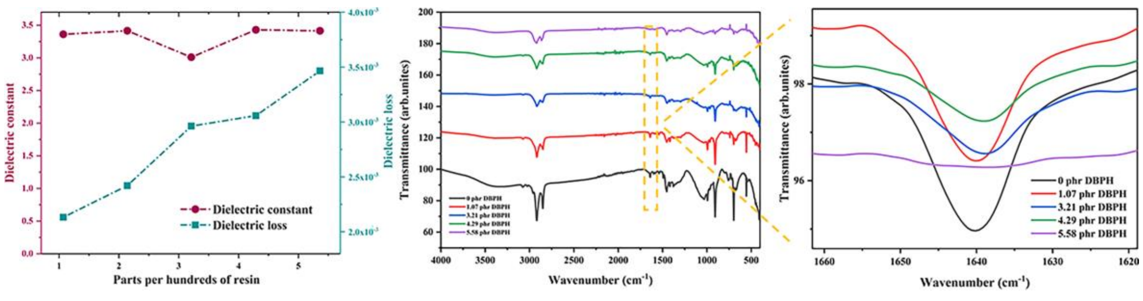

Figure S1. (a) D_k and D_f of samples with a different content of DBPH; (b) FTIR spectrum of samples with a different content of DBPH; (c) partial enlargement of the FTIR spectrum of samples with a different content of DBPH, between 1660 cm^{-1} and 1620 cm^{-1}.

Table S1. Thickness of the different samples.

**Samples**	**Thickness (mm)**		
C0	0.897	0.939	0.871
C5	0.841	0.822	0.853
C10	0.759	0.716	0.773
C15	0.773	0.794	0.751
C20	0.844	0.835	0.852
S0	0.847	0.843	0.860
S1	0.875	0.877	0.873
S2	0.925	0.935	0.915
S3	0.829	0.831	0.826
S4	0.918	0.912	0.924
S5	0.932	0.941	0.921
